# Impact Damage Evaluation in Composite Structures Based on Fusion of Results of Ultrasonic Testing and X-ray Computed Tomography

**DOI:** 10.3390/s20071867

**Published:** 2020-03-27

**Authors:** Andrzej Katunin, Angelika Wronkowicz-Katunin, Krzysztof Dragan

**Affiliations:** 1 Department of Fundamentals of Machinery Design, Silesian University of Technology, Konarskiego 18A, 44-100 Gliwice, Poland; angelika.wronkowicz-katunin@polsl.pl; 2 Division of Airworthiness, Air Force Institute of Technology, Ks. Bolesława 6, 01-494 Warsaw, Poland; krzysztof.dragan@itwl.pl

**Keywords:** barely visible impact damage, information fusion, non-destructive testing, ultrasonic testing, X-ray computed tomography.

## Abstract

Barely visible impact damage (BVID) is one of the most dangerous types of structural damage in composites, since in most practical cases the application of advanced non-destructive testing (NDT) methods is required to detect and identify it. Due to its character of propagation, there are minor signs of structural damage on a surface, while the internal damage can be broad and complex both in the point of view of fracture mechanisms and resulting geometry of damage. The most common NDT method applied e.g., in aircraft inspections is ultrasonic testing (UT), which enables effective damage detection and localization in various environments. However, the results of such inspections are usually misestimated with respect to the true damage extent, and the quantitative analysis is biased by an error. In order to determine the estimation error a comparative analysis was performed on NDT results obtained for artificially damaged carbon fiber-reinforced composite structures using two UT methods and X-ray computed tomography (CT). The latter method was considered here as the reference one, since it gives the best spatial resolution and estimation accuracy of internal damage among the available NDT methods. Fusing the NDT results for a set of pre-damaged composite structures with various energy values of impact and various types of impactor tips applied for introducing damage, the evaluation of estimation accuracy of UT was possible. The performed analysis allowed for evaluation of relations between UT and X-ray CT NDT results and for proposal of a correcting factor for UT results for BVID in the analyzed composite structures.

## 1. Introduction

Composite structures are characterized by high resistance to loads oriented along fiber direction. Unlike metals, composites allow easier forming of non-ruled surfaces with large sizes. This creates a possibility of producing relatively light components with a low number of subcomponents. Another advantage is the fatigue characteristics of composite structures, which is better than in the case of metal structures. However, the experience from the operation of aircraft components made of composite structures pointed to different sources of damage than in the case of metals. In the case of the metal structures, the basic sources of damage are the effect of variable load cycles, whereas in composites the damage is caused mainly by mechanical impacts during maintenance [1,2]. The character of damage in composite structures caused by impacts depends on the velocity and the energy of the impact. This work focuses on a low velocity impact damage. Under this term the damage is understood as caused by objects striking at low speeds and relatively low energy (<15 J). The low velocity impact damage can cause the occurrence of barely visible impact damage (BVID). This term is understood as the internal defects of the composite without any damage indication by a visual evaluation [3]. The internal damage is caused mainly by compression and interlaminar shear stresses [4]. In effect, delamination and matrix cracks occur. The size and shape of delamination depend on the energy and velocity of the impactor as well as a method of fixing of the composite structure. Failure modes that occur during the impact may have several types and their presence is very often impossible to be proved without the application of a proper non-destructive testing (NDT) method [5].

The most popular method used for a composite inspection is ultrasonics, where a signal can be transmitted in a material and received in a few different ways, but in every case the amplitude or/and time-of-flight are evaluated. Other approaches enabling damage detection on the basis of the emitted sound wave are methods such as: tap-test, mechanical impedance analysis (MIA), resonant method or pitch-catch [5]. In all these methods the principle of operation is similar. The acoustic signal is emitted and then received. The responses from a damaged and an undamaged area are different [6,7]. The differences between the particular methods result from an applied measurement frequency and a construction of the measuring sensor.

Basically, the ultrasounds are the acoustic elastic waves with frequencies greater than the audible to human hearing (the so-called hearing threshold above 16–20 kHz). The ultrasonic testing (UT) is the most often used NDT methods of composite materials [8,9,10]. The method allows the use of many measurement configurations, such as: through-transmission method, pulse-echo method and the use of surface waves [11,12,13]. The UT method, by measuring the time of flight of the acoustic wave through the tested composite element and the amplitude of the signal, allows for the assessment of a type, size and depth of damage [14]. Measurements using the ultrasonic method require the use of appropriate measuring equipment for recording the signals. The processing of the measured signals allows for the determination of many characteristics of a tested material, such as attenuation [15], fiber direction [16] and distribution of damage [17]. The UT method allows for the detection of a wide range of damage types, however, in many cases it requires a proper interpretation of the measured signals using data processing techniques [18]. A limitation of this method is the necessity of using coupling agents, as well as difficulties when examining elements that are very thin or thick and/or have a complex shape. The UT method applied for composites is used mainly in two modes: the pulse-echo and through-transmission.

A radiographic testing (RT) is an NDT method of composites using a short wavelength electromagnetic radiation (X-ray or gamma ray). Variations in material density, thickness, composition, or a presence of defects result in absorbing different amounts of the penetrating radiation by different parts of a tested component. The unabsorbed radiation that passes through the part is recorded on a radiographic film or a photosensor, and the result is shown on the film or electronic radiation detectors [19]. The method is able to detect flaws of composites, such as trans-laminar cracks, foreign inclusions, porosity, voids, fiber folds or wrinkles. In conventional RT, de-lamination can be also detected if is not perpendicular to the X-ray beam [20]. Currently, this method is increasingly being replaced by computed radiography (CR), where the traditional film is replaced with a photostimulable phosphor (PSP) plate. Next, a more advanced technique, with enhanced defect detection and location capabilities, is X-ray computed tomography (X-ray CT). This method requires rotation of the object (or the source and detector) up to 360°. Unlike radiography, X-ray CT involves the generation of cross-sectional views (virtual slices of the object) instead of a planar projection. It is an excellent technique for a 3D visualization of a part’s interior features. This method allows also for easy quantification of porosity in composites [21]. However, the biggest limitation is the size of tested elements, which must fit in a tomograph chamber.

As one can notice, there is not only one chosen successful NDT method used for damage detection in fiber reinforced polymers. The use of each method is associated with limitations and restrictions not only due to the material properties and geometrical constraints but also with failure mode type and location. The awareness of multiple failure mode presence helps with a proper selection of the best tailored NDT solution for a tested case. An option is the use of multiple methods and damage analysis, but such an approach is costly and time consuming. Moreover, the presence of uncertainty in estimation of damage extent is a serious problem, which may limit practical applications of a given NDT method to qualitative analysis only. One of the methods used to overcome such deficiencies is the application of fusion techniques applicable to results of NDT using different methods. The use of data fusion may accelerate the damage characterization as well as increase the diagnostics capabilities. This approach is well known from previous studies available in literature. The authors of [22] applied fusion of NDT results obtained using three different NDT methods: acoustic emission, digital image correlation (DIC) and infrared thermography (IRT), which allowed them to enhance a damage quantification procedure in glass fiber-reinforced epoxy laminated structures. Daryabor and Safizadeh [23] presented an approach for fusion of UT and IRT NDT results for the improvement of the inspection capabilities of carbon/epoxy patches bonded to an aluminum plate. Yuan and Wang [24] proposed, in turn, a combination of IRT and DIC for the estimation of compressive failure properties of tested structures. A fusion of inductive IRT and UT NDT results were used by Xiao et al. [25] for the improvement of damage detectability in aluminum alloy structures. The authors of [26] fused the results of two other NDT methods: IRT and shearography, which significantly reduced the measurement error and improved quantification ability of impact damage in tested carbon fiber-reinforced polymeric (CFRP) structures. In this approach, the authors used also UT in a C-Scan mode for validation purposes. Several approaches of NDT data fusion were implemented also in diagnosing of civil concrete structures [27,28,29]. Although, several applications of fusion of results of various NDT methods are presented in literature, this approach in structural inspection remain open for new solutions and has a great potential in the enhancement of ability of damage detection and quantification. This motivated the authors of the following study to apply such a concept in the evaluation of accuracy of estimation of damage extent in composite structures with BVID.

Due to the fact that UT results usually present under- or over-estimated damage with respect to true damage [30], the damage extent estimation is difficult, since this misestimation introduces uncertainty in the evaluation process. Therefore, it is important to characterize damage mechanisms typical for CFRP structures subjected to low-velocity impact loading depending on specific conditions of such loading as well as to evaluate the amount of misestimation which allows for a minimization of errors in damage extent estimation based on solely UT results. For this purpose, a fusion of the obtained UT results in B-Scan and C-Scan modes were fused with X ray CT results of CFRP specimens pre-damaged using various impactors and various impact energies. The X-ray CT NDT method was used here as a reference due to its ability of the reconstruction of internal damage in three dimensions, high sensitivity to various elementary types of damage (such as cracks, delamination, debonding, etc.) as well as much higher spatial resolution with respect to UT results. The results of fusion allowed for the evaluation of an amount of misestimation in the BVID identification in tested CFRP structures and development of a measure, which enables a description of this misestimation using a correcting factor introduced in this study. The presented approach is useful especially in correcting the damage extent in the considered specimens obtained directly from UT, and further evaluation of a structural residual life, including its prediction for structures being in operation as well as modeling the structural life in CFRP structures subjected to BVID.

## 2. Specimens and Experiments

### 2.1. Specimens Preparation and Introduction of Impact Damage

**Figure 1:**
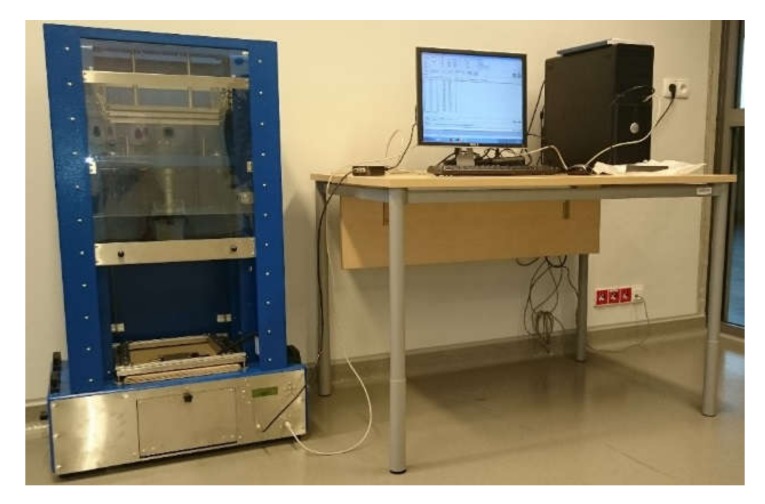
A drop weight testing machine for introducing impact damage in composites.

For the presented experiments, specimens made of CFRP structures with the dimensions of 100 × 100 × 2.5 mm (length × width × thickness) were applied. The specimens were produced by the Dexcraft s.c. (Helenów, Poland) using epoxy resin LG 700 manufactured by GRM Systems (Olomouc, Czech Republic) reinforced with 10 layers (with the same layup) of a 2 × 2 twill weave Toray T300 (Toray Composite Materials America, Inc., Tacoma, WA, USA) carbon fabric of 200 g/cm^2^ in the VARTM resin infusion technology. Then, various scenarios of impact damage were introduced to the specimens using a drop weight testing machine presented in Figure 1. The detailed description on the testing machine can be found in [31]. In total, 16 BVID scenarios were introduced. All the resulting damage cases were qualified as BVID according to [32], which states that BVID is damage which is not visible from a distance of 1524 mm (5 ft) using typical lighting conditions. The obtained damage cases are further in this article called by their symbols consisting of the impactor shape designation (C—for a 22 mm, D—for a 16 mm, and E—for a 10 mm diameter of its hemispherical tip) and the energy of impact—in the range of 5−20 J.

### 2.2. Non-Destructive Testing of Specimens

**Figure 2:**
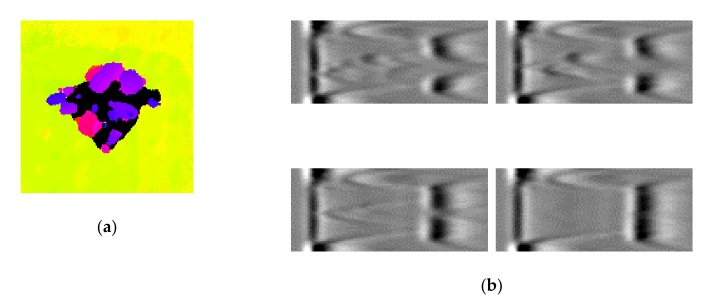

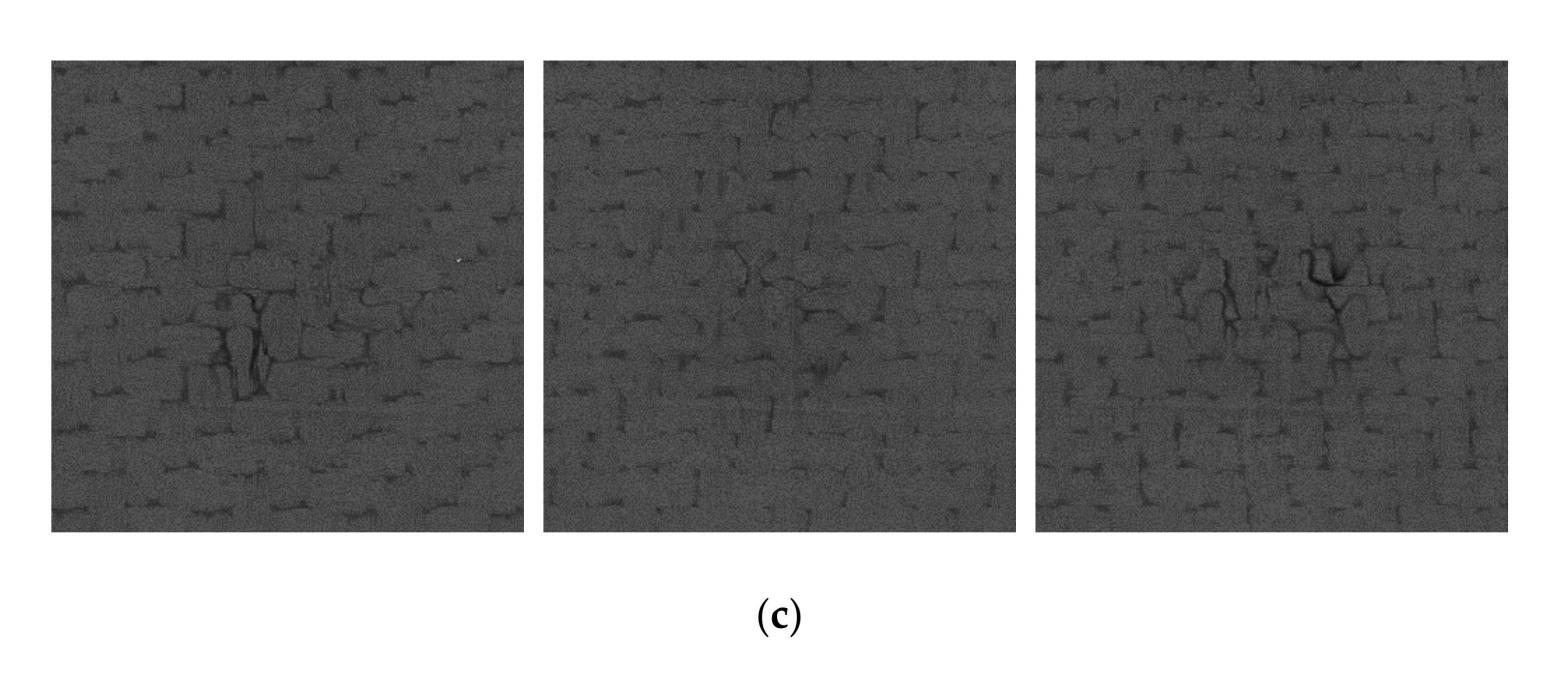
Fragments of raw non-destructive testing (NDT) results obtained for the selected damage case (E17.5): the ultrasonic C-Scan (**a**); selected sections of the ultrasonic B-Scan (**b**), and the X-ray computed tomography (CT) scan (**c**).

The next step was to perform NDT using three methods and acquire the resulting data containing information about the detected damage to be processed in the next steps of this study. Namely, the specimens were scanned using a pulse-echo UT method in the C-Scan mode (see an exemplary result in Figure 2a) and a phased-array UT method in the B-Scan mode (Figure 2b), as well as the X-ray CT method (Figure 2c). The applied technical parameters of these inspection methods were described in [33].

## 3. Fusion of NDT Results

### 3.1. Fusion Algorithm

The developed fusion algorithm steps are as follow. Firstly, the raw data acquired during NDT of the composite specimens, namely the ultrasonic C-Scans, B-Scans and X-ray CT scans (as the reference data), were individually processed in order to detect the BVID areas and obtain their reconstructed 3D binary images. For this purpose, previously developed methods by the authors, described in detail in [33], were applied. It should be highlighted that the methods of processing of each type of the NDT data are different and, in the above cited paper, several approaches were proposed. For this study, the authors applied the method of processing: the X-ray CT scans based on the texture-based image segmentation; the C-Scans based on the MBP-based image segmentation and 3D reconstruction; and B-Scans based on the histogram-based image segmentation that enables an automatic detection of the binarization threshold value. The algorithms of X-ray CT scans as well as B-Scans processing were additionally improved by masking the data by the area around a contour of the biggest object detected in a planar view of the 3D image (the view of impact damage), which allowed for a removal of noise around the damage.

**Figure 3:**
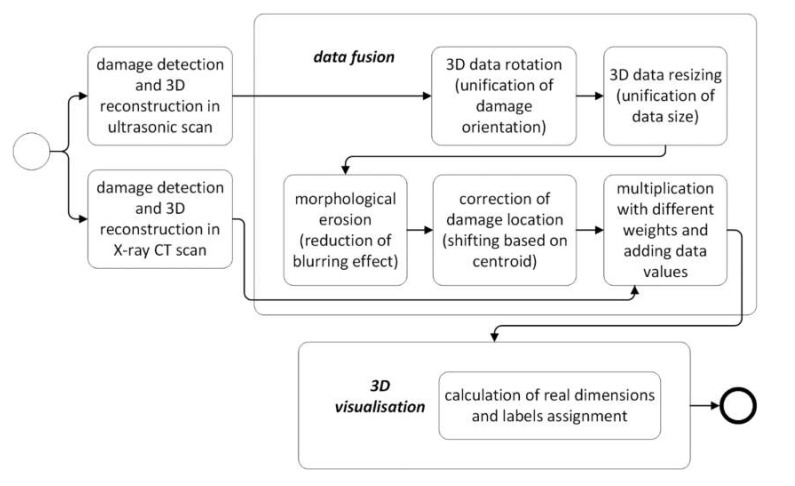
A flowchart of the algorithm of fusion of ultrasonic B-Scans/C-Scans with X-ray CT scans.

**Figure 4:**
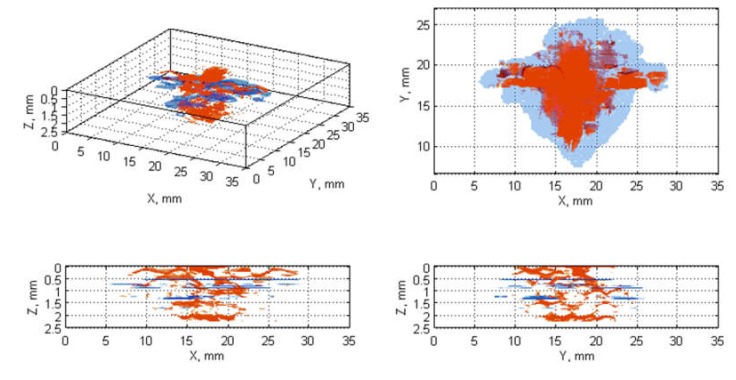
Views of fused damage detected in the X-ray CT scans (red color) and the ultrasonic C-Scan (blue color) for the case E17.5.

**Figure 5:**
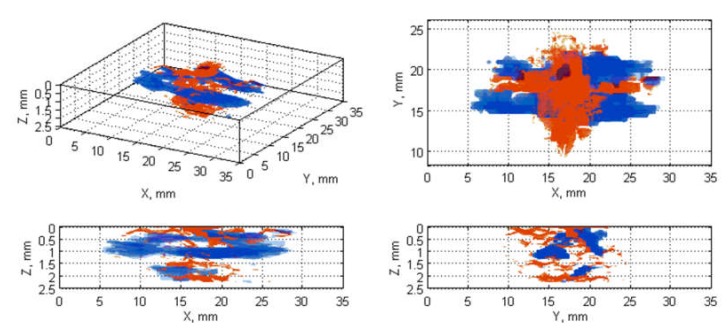
Views of fused damage detected in the X-ray CT scans (red color) and the ultrasonic B-Scan (blue color) for the case E17.5.

The next stage was the fusion of the damage regions extracted from the ultrasonic data (B-Scans/C-Scans) with the ones from X-ray CT scans. This was performed by several steps. At first, the damage orientation in all NDT data types had to be unified by their appropriate rotating, which resulted from scanning the specimens in different ways during each of the NDT tests. Then, the ultrasonic data was resized (enlarged by a ratio calculated based on known pixel resolution of each data type) to have the same resolution as the tomographic scans. After that, a morphological erosion was applied to the ultrasonic data in order to reduce a blurring effect produced as a result of the resizing step. Next, a correction of damage location was introduced by shifting the binary data results to have a common centroid in the planar damage views. Finally, values of the resulting pairs of matrices, that were to be fused, were added after a multiplication with weights different for X-ray CT and UT data, which enabled displaying them as different colors in the visualization of the fusion results. The flowchart for the described algorithm is presented in Figure 3, while the exemplary results being the output of this algorithm for various pairs of NDT datasets, namely C-Scan and X-ray CT, B-Scan and X-ray CT scans are presented in Figure 4 and Figure 5, respectively.

### 3.2. Results of Fusion

All the collected NDT results were processed using the above described image processing algorithms. The obtained images of the fused damage areas for an exemplary case (E17.5) are presented in Figure 4 and Figure 5, respectively for the results of processing the X-ray CT scan fused with the C-Scan and with the B-Scan.

When visually analyzing the obtained images one can observe that the studied BVID is very well detectable using both the applied ultrasonic testing techniques. The identified damage areas are comparable with the reference ones extracted from the X-ray CT scans, but the damage shape is more accurate in C-Scans (see the X-Y views in Figure 4), whereas in B-Scans it is distorted (Figure 5).

The observed differences are a result of sensitivity of particular methods to particular elementary types of damage occurred in BVID. Analyzing the exemplary case presented in Figure 4 and Figure 5, one can observe that both UT methods did not detect the surface indentation and resulting matrix cracks and debonding, which is especially well visible on the front views of the mentioned figures. The reason of the insensitivity of UT methods to surface and subsurface damage results from so-called dead zone, which is a zone in a thickness direction of a tested structure in which the echo from the subsurface damage is covered by the initial pulse of the transmitted ultrasonic wave. On the other side, it can be noticed that some parts of damage near the bottom surface of the specimens were also not detected using UT methods, which is due to a reduction of heights of echoes from these damage regions resulting from the distance traveled by the ultrasonic wave as well as resulting from a reflection of the most part of this wave from delaminations located above. More details on these effects one can find in [30]. However, comparing the results obtained for B- and C-Scans one can state that the dead zone in the case of B-Scans is lower with respect to C-Scans, which has a confirmation in all other obtained results. The whole sets of the obtained results from the comparison of X-ray CT scans with B- and C-Scans, respectively, are presented in Appendix A.

In order to analyze the results quantitatively, a correlation measure is proposed, and the results of its calculation are presented in the next sections.

### 3.3. Analysis of Damage Mechanisms and Their Detectability

The acquisition of the NDT results using various methods, in particular X-ray CT as the reference method, for different impactor shapes and impact energy values made it possible to evaluate the influence of these properties on the resulting BVID as well as its detectability with ultrasonic NDT methods. Since the X-ray CT method allowed obtaining the most accurate results of internal structural damage after impact loading, further analysis of damage mechanisms was performed based on this type of NDT data.

From the preliminary observations one can notice that the resulting BVID in the considered cases has no classical geometry of a fracture mechanism (the pine tree distribution damage—see [33] for more details), which may result from a relatively thin structures subjected to impact loading (the distribution of internal stresses between the composite layers did not allow for a full development of this type of damage distribution) as well as boundary conditions of the impacted structures (fixing on side surfaces and support on the bottom edge and a part of the bottom surface—ca. 5 mm from the edges—could redistribute the stress state). However, tendencies and regularities in fracture mechanisms depending on the type of impactor and impact energy can be observed.

**Figure A1:**
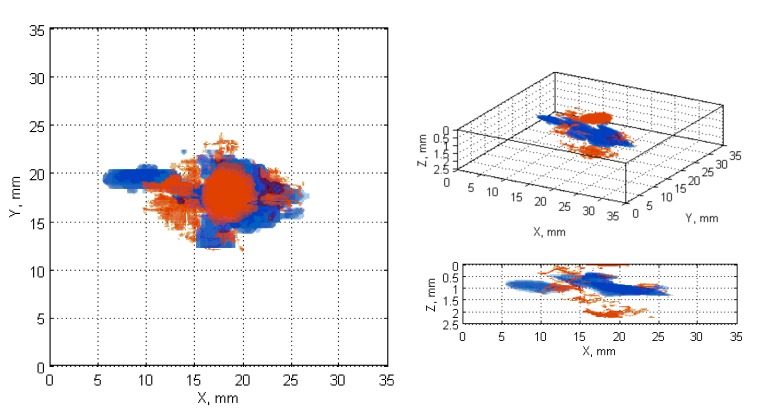

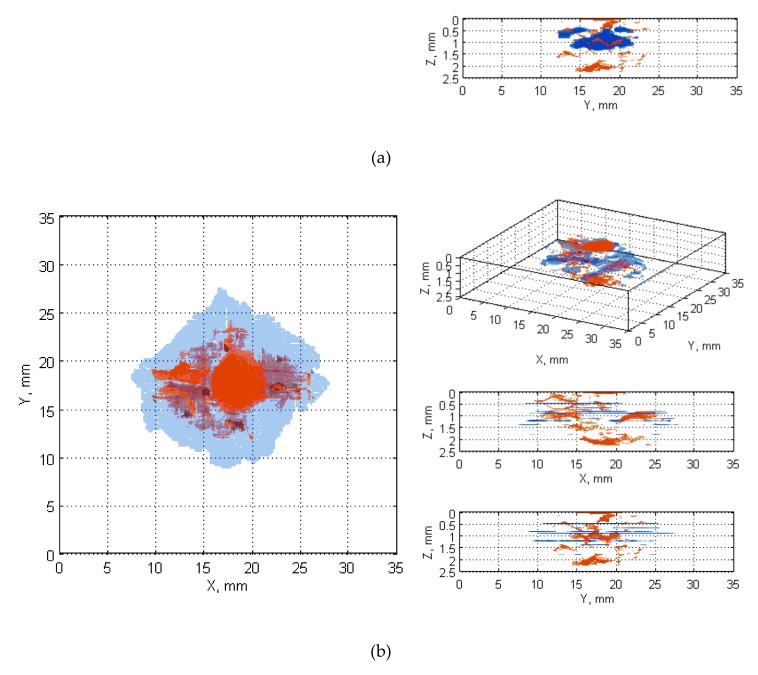
Fused damage detected in the X-ray CT scans (red color) and the ultrasonic (blue color) B-Scan (**a**) and C-Scan (**b**) for impactor C with impact energy of 15 J.

**Figure A2:**
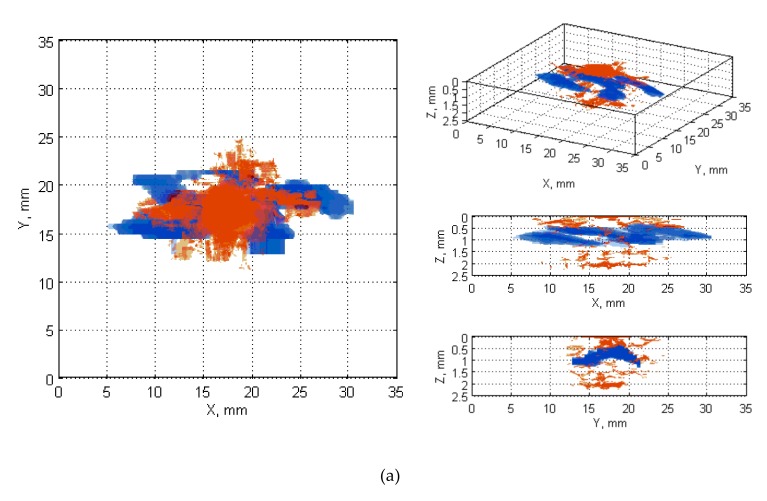

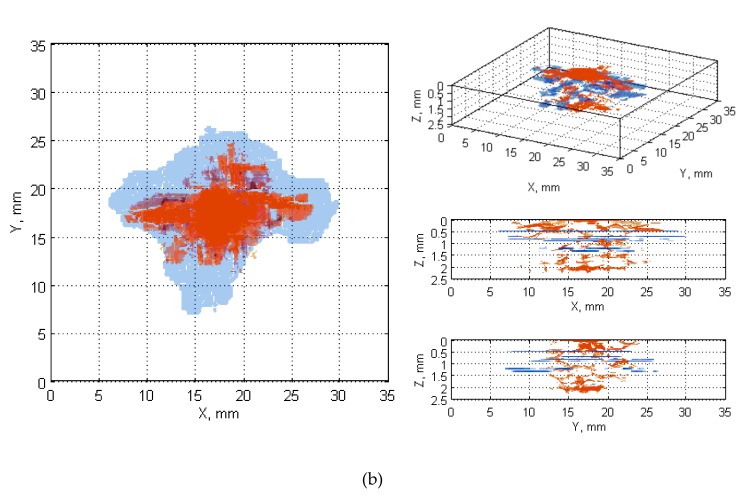
Fused damage detected in the X-ray CT scans (red color) and the ultrasonic (blue color) B-Scan (**a**) and C-Scan (**b**) for impactor C with impact energy of 17.5 J.

**Figure A3:**
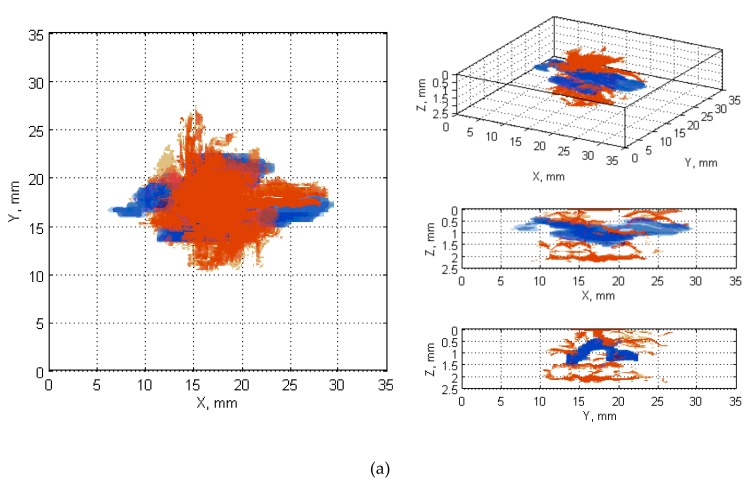

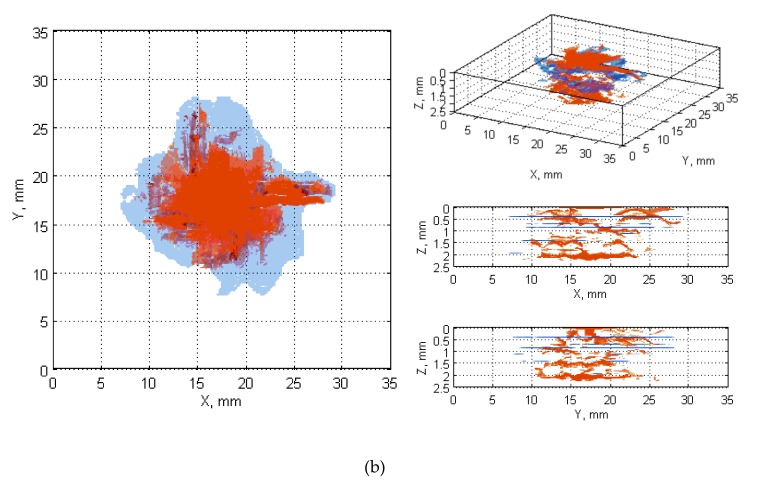
Fused damage detected in the X-ray CT scans (red color) and the ultrasonic (blue color) B-Scan (**a**) and C-Scan (**b**) for impactor C with impact energy of 20 J.

**Figure A7:**
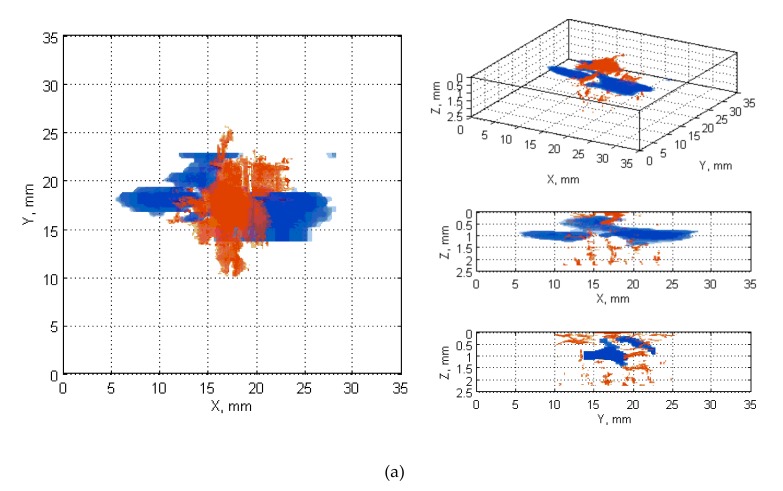

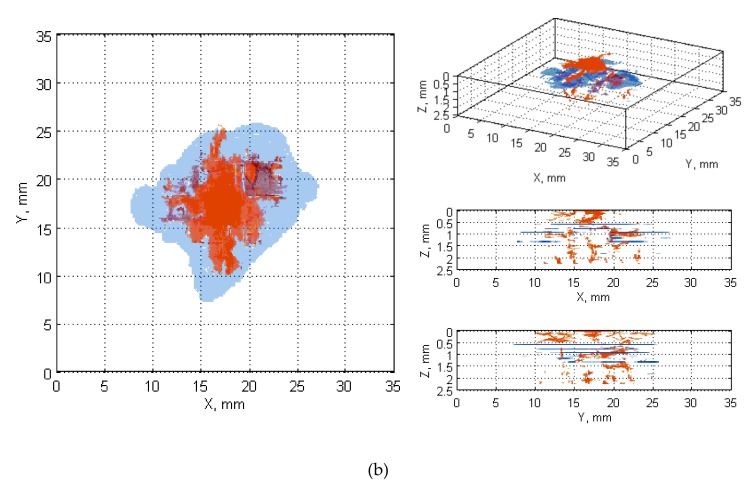
Fused damage detected in the X-ray CT scans (red color) and the ultrasonic (blue color) B-Scan (**a**) and C-Scan (**b**) for impactor D with impact energy of 15 J.

**Figure A8:**
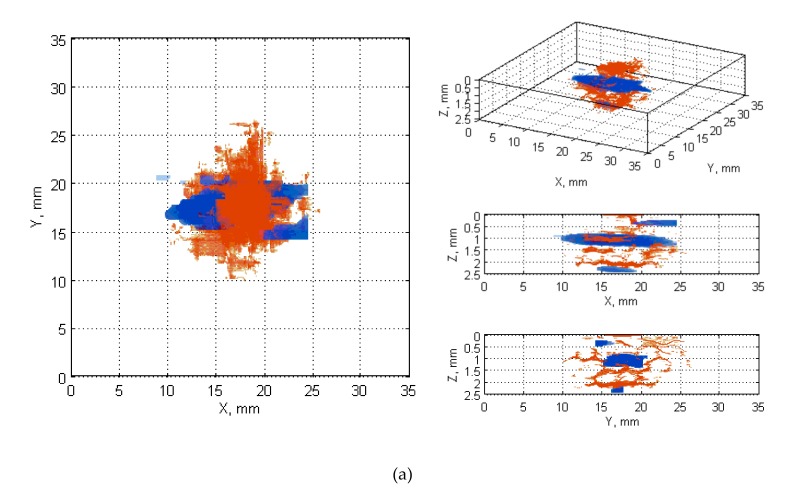

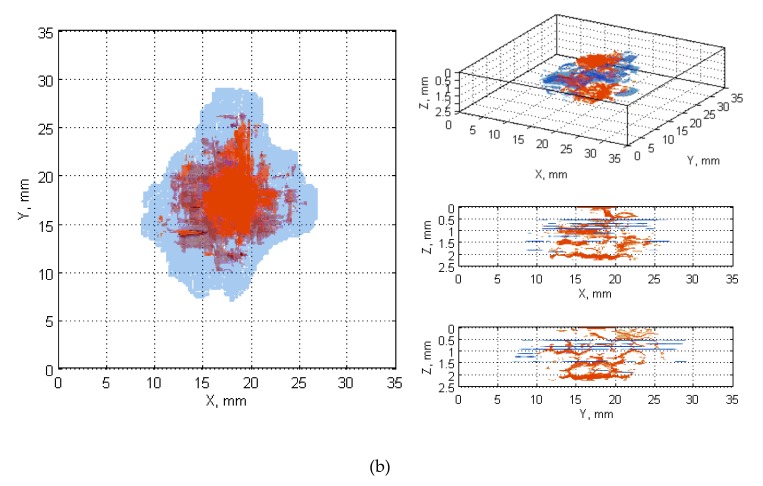
Fused damage detected in the X-ray CT scans (red color) and the ultrasonic (blue color) B-Scan (**a**) and C-Scan (**b**) for impactor D with impact energy of 17.5 J.

**Figure A9:**
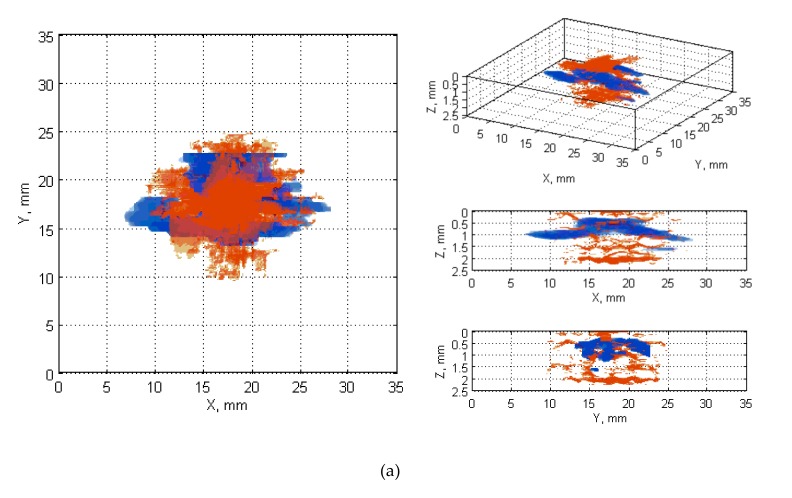

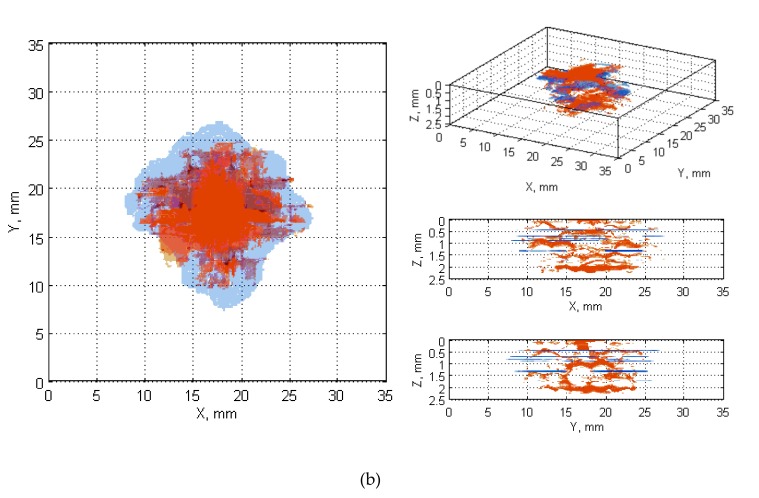
Fused damage detected in the X-ray CT scans (red color) and the ultrasonic (blue color) B-Scan (**a**) and C-Scan (**b**) for impactor D with impact energy of 20 J.

**Figure A14:**
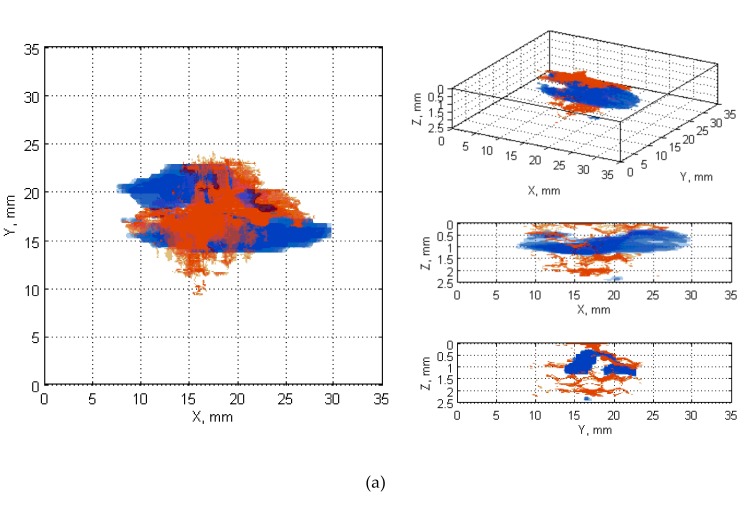

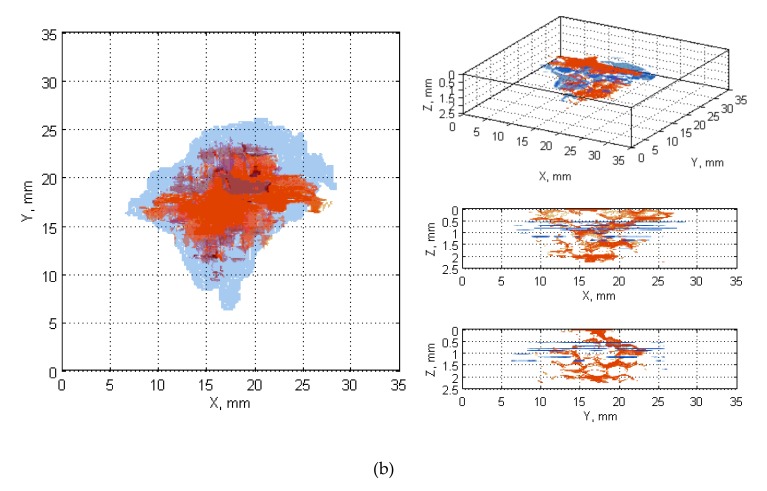
Fused damage detected in the X-ray CT scans (red color) and the ultrasonic (blue color) B-Scan (**a**) and C-Scan (**b**) for impactor E with impact energy of 15 J.

**Figure A15:**
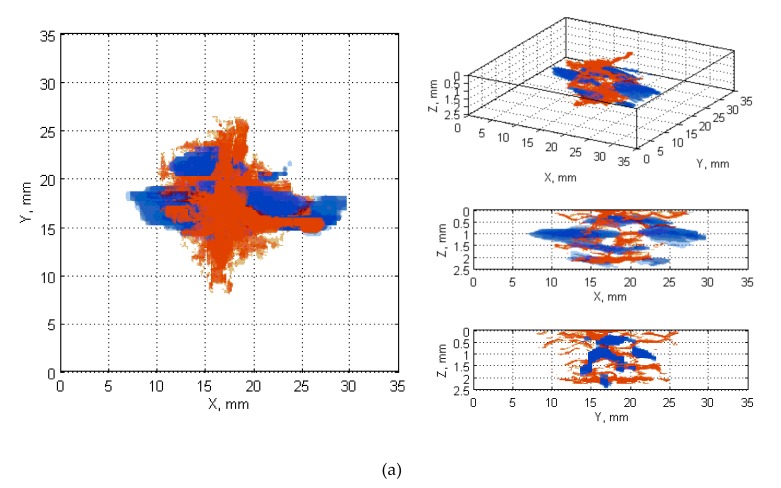

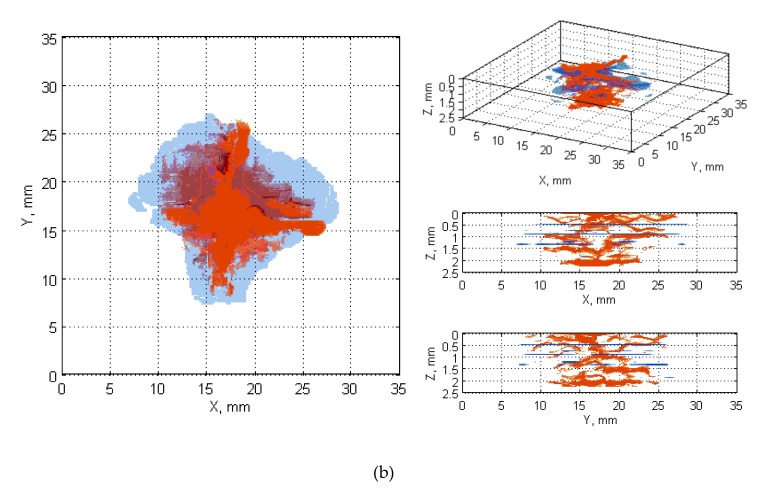
Fused damage detected in the X-ray CT scans (red color) and the ultrasonic (blue color) B-Scan (**a**) and C-Scan (**b**) for impactor E with impact energy of 20 J.

Comparing the results obtained for the impacted structures with various impactors and same energies (3 sets of 15 J, 17.5 J, and 20 J—see Appendix A), one can conclude that with a decrease of the radius of a hemispheric tip of an impactor (see Section 2.1 for more details) the area of surface damage also decreases, which is obvious due to higher stress concentration in the impact location. However, with the decrease of this radius one can observe increasing the density of internal damage. This tendency is observable for all 3 sets, namely for impact energy of 15 J (cf. Figure A1, Figure A7 and Figure A14), 17.5 J (cf. Figure A2, Figure A8, Figure 4 and Figure 5), and 20 J (cf. Figure A3, Figure A9 and Figure A15).

In the case of the increase of impact energy for particular impactors considered in this study one can observe a proportional increase of the density of resulting internal damage with spreading translaminar delamination and cracks resulted from shear stresses during impact loading. The increase of the density of structural damage in bottom layers is also proportional to the applied impact energy, which is a direct result of increasing the magnitude of shear and bending stresses with the increase of impact energy.

The developed fusion algorithm allowed also for the evaluation of the detectability of BVID using UT methods considering the reference NDT data obtained from X-ray CT. The performed qualitative analysis shows a tendency similar to the described one for the analysis of damage progression based on X-ray CT results. In particular, for B-Scans one can observe both spreading the damage locations both in plane as well as in transversal directions during increasing the impact energy. For C-Scans this tendency is not so obvious due to technical limitations of the method (e.g., dead zone and distance-amplitude effects described in Section 3.2), however, in general, similar behavior is observable. In this scanning mode one can point an attention on the ability of proper reflection of damage in the thickness direction, which has a crucial importance from the point of view of estimation of structural residual strength and stiffness. In order to evaluate this influence in a quantitative way the estimation accuracy was performed, which was described in detail in the next section.

### 3.4. Evaluation of Estimation Accuracy of BVID Based on UT

**Figure 6:**
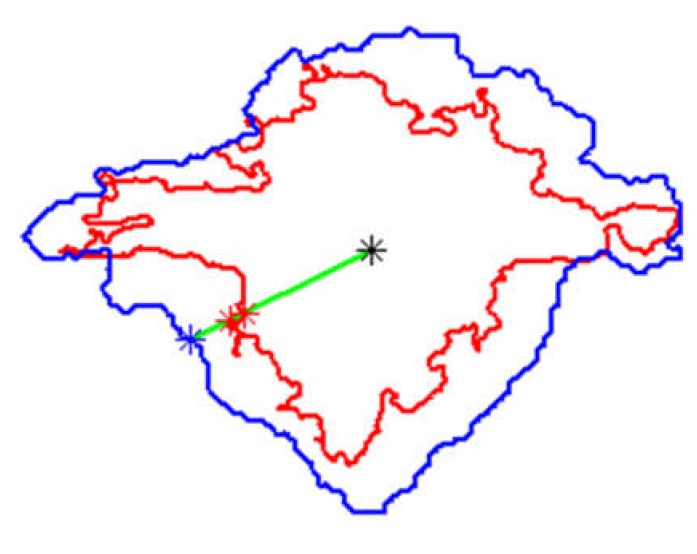
A method of the distance calculation between an exemplary boundary point of the C-Scan–case E17.5 (marked blue) and X-ray CT scan (red) using a centroid (black); explanation in the text.

In order to evaluate the damage estimation accuracy based on ultrasonic testing results with respect to the tomographic ones, a measure based on distances between damage boundaries was elaborated. Namely, for each point of a boundary of BVID (extracted from its planar view) detected in a C-Scan/B-Scan, a distance is measured to the point of damage boundary extracted from an X-ray CT scan with the assumption that both points belong to the same curve also passing through the damage centroid. If there is more than one point of the X-ray CT boundary crossing this curve, the outermost one is considered. The example is presented in Figure 6, where red colors are for boundary and points of the X-ray CT, blue colors for the C-Scan, and black point represents the centroid location. Moreover, the sign of the distance value is determined, which returns a quantitative data about damage underestimation or overestimation based on the UT results. Such an approach returns more objective results than in the case of calculating this distance just to the nearest point, since, here, the distances are calculated in all the clockwise directions.

**Figure 7:**
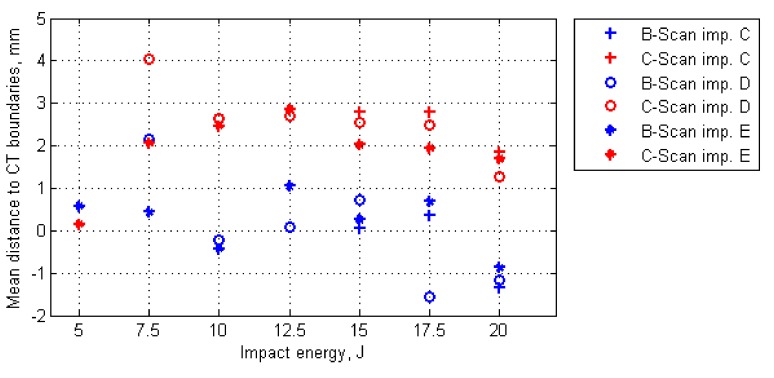
Mean distances between all points of damage boundaries detected in ultrasonic B-Scans/C-Scans and X-ray CT scans (for considered impact energies and shapes of impactors).

**Figure 8:**
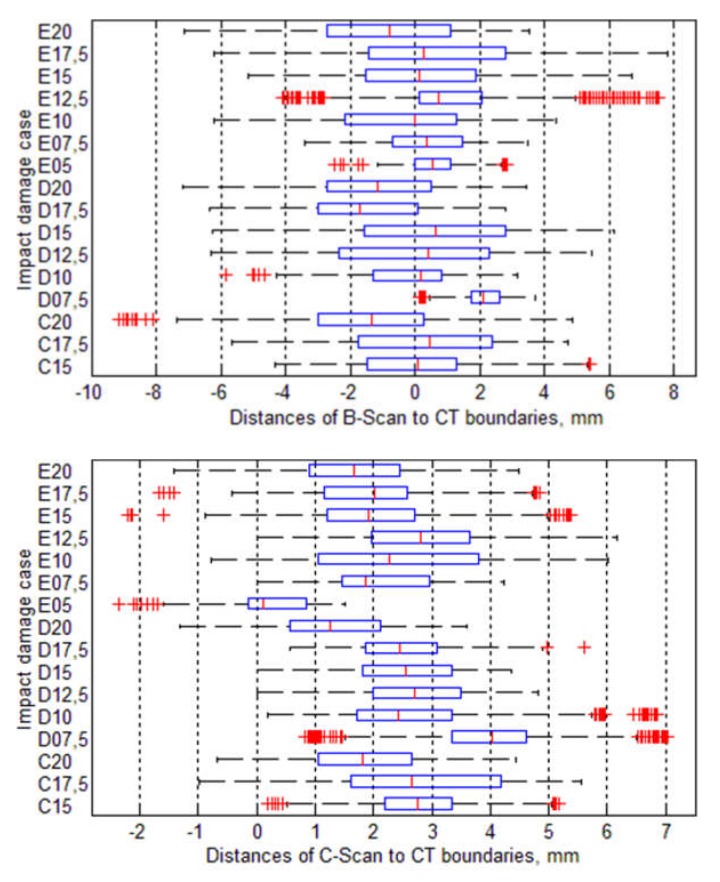
Statistics of the distances between damage boundaries (the boxes’ central marks denote median values, boundaries of the boxes represent 25th and 75th percentiles, whiskers indicate extreme data points, and red signs + are the singular data points beyond the whiskers).

Mean values of the calculated distances for all the considered cases are presented graphically in Figure 7. The initial analysis of these results could indicate that B-Scans bring more exact information about damage since the mean distances are around 0 mm (the exact average for all the damage cases is 0.05 mm), whereas for C-Scans they are mostly around 2−3 mm. However, when analyzed other statistics of the distances’ values (see Figure 8), it can be noticed that the range of distances’ values for B-Scans is much wider (between −10 mm and +8 mm) than for C-Scans (where it is between −2 mm and +7 mm). This means that although the damage extent detected in C-Scans is mostly overestimated (on average by 2.27 mm), the shape of damage is regularly close to the real one (i.e. extracted based on X-ray CT). This stays in contrast to B-Scans, where damage contours are either overestimated or underestimated, i.e., the shape is irregularly different from the real one. These relations can be also observed in the images of damage views, as in the exemplary case presented in Figure 4 and Figure 5 (see especially the planar X-Y views).

Summarizing the above presented results for the investigated damage cases and used NDT techniques, the estimation accuracy of BVID based on ultrasonic B-Scans turned out to be very high when considered only the mean distances between the detected damage contours’ points to the reference data (X-ray CT scans), which can be related also to the possibility of calculation of a proper surface area of damage. However, taking into account also the shape of detected damage, it can be stated that C-Scans brought more accurate information, but with the damage overestimation of about 2.27 mm in each direction. These damage size differences can be explained by many factors influencing on the measurement uncertainty using ultrasonic testing techniques, such as an applied transducer frequency and a beam diameter, which was described in detail in a previous authors’ work [30].

In further experiments based on the examined composite structures and considered BVID scenarios, the authors plan to apply a correcting factor of 2.27 mm for ultrasonic C-Scans (for a removal of the external areas of damage along its contour), which will give more reliable information about real damage extent while maintaining its proper shape. Using such corrected C-Scans instead of the obtained B-Scans for the planned purposes of damage modeling and estimating the residual strength of the composite structures will provide better results due to the good shape reflection, which may be important in the context of the direction of damage propagation.

## 4. Conclusions

In the following study, the authors presented the results of fusion of non-destructive testing results of CFRP structures with BVID using two modes of UT, B- and C Scans, as well as X-ray CT used here as the reference method. The proposed fusion approach allowed to characterize the resulting damage after low-velocity impact loading taking into consideration parameters of this loading, estimate the detectability of impact damage using both UT methods basing on fusion of UT and X-ray CT results, and finally to evaluate misestimation of B- and C-Scans, which are commonly used in inspections of composite structures, in particular, aircraft structures, with respect to X-ray CT scans, which provide much higher resolution and sensitivity to damage than the mentioned UT methods. The importance of determination of this misestimation results from two factors of the practical inspection procedures: first, the X-ray CT method is limited by the size of tested objects and the tests can be performed in laboratory conditions only; and second, the cost of testing using UT is significantly lower than when using the X-ray CT method. The results of the performed tests and further image processing of the obtained results allowed for determination of the correcting factor for UT testing, which allows to represent the resulting BVID in the tested CFRP specimens within this method in a more realistic manner. It should be highlighted that, due to the necessity of individual selection of UT techniques and parameters for each tested composite structure, such a correcting factor should be calculated using the presented method individually for different types and dimensions of a composite with BVID being evaluated. This also applies to the situation of investigating different damage scenarios.

## Figures and Tables

**Figure A4:**
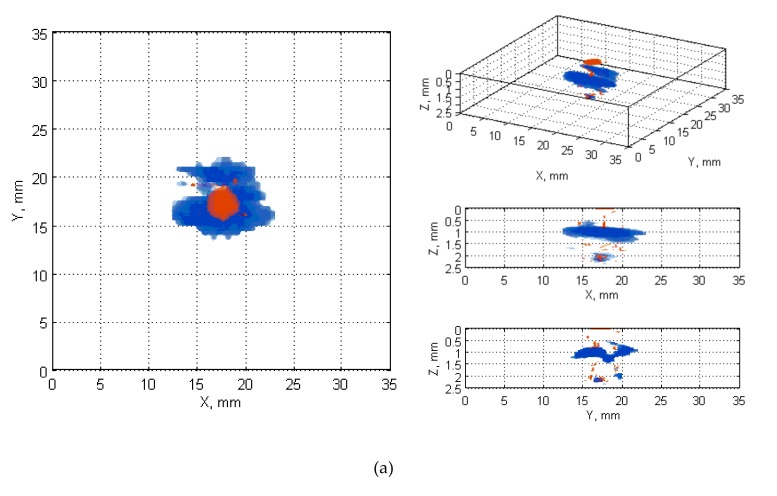

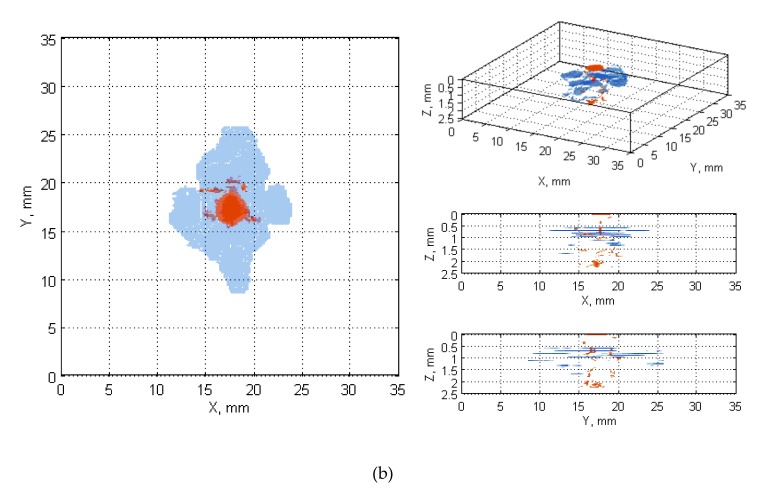
Fused damage detected in the X-ray CT scans (red color) and the ultrasonic (blue color) B-Scan (**a**) and C-Scan (**b**) for impactor D with impact energy of 7.5 J.

**Figure A5:**
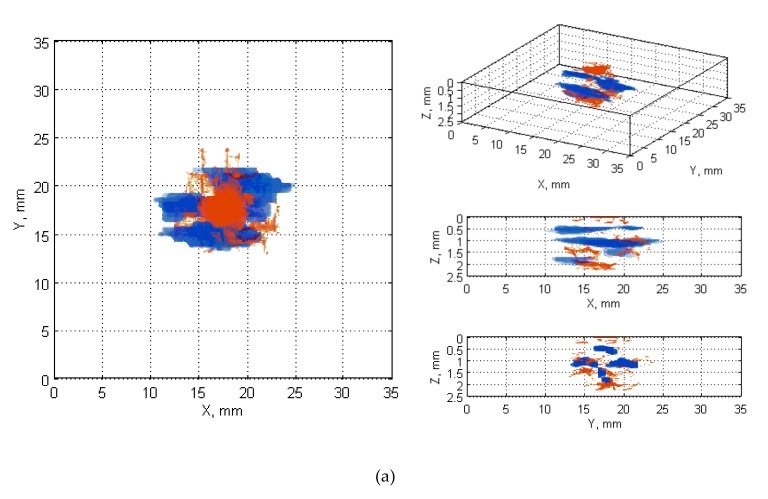

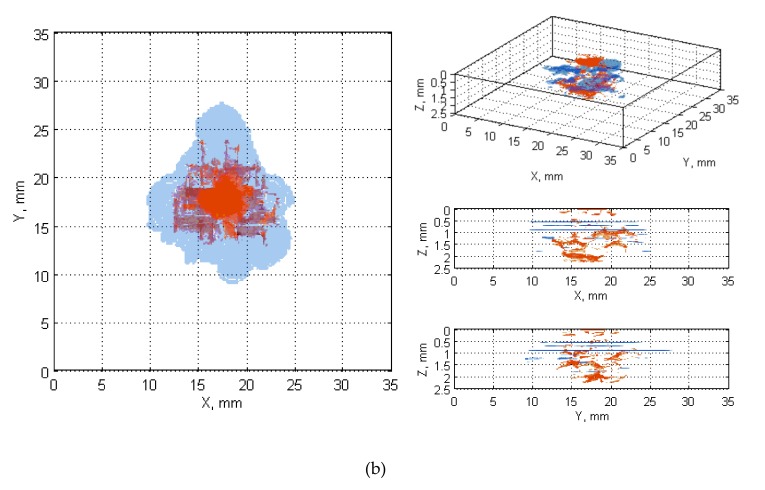
Fused damage detected in the X-ray CT scans (red color) and the ultrasonic (blue color) B-Scan (**a**) and C-Scan (**b**) for impactor D with impact energy of 10 J.

**Figure A6:**
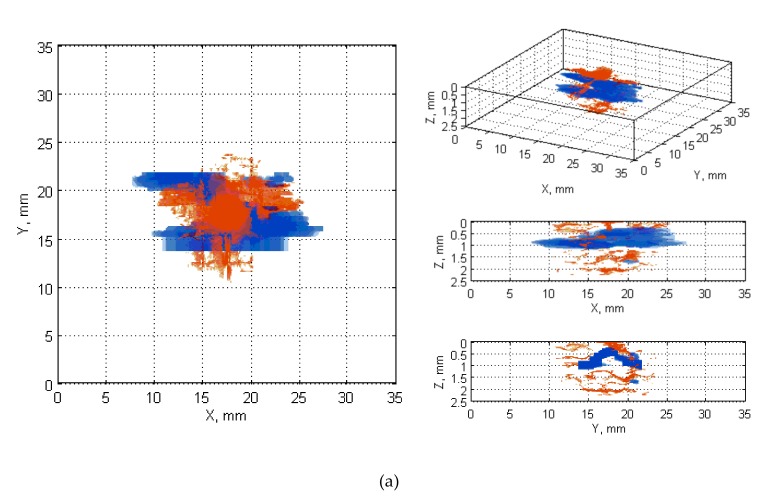

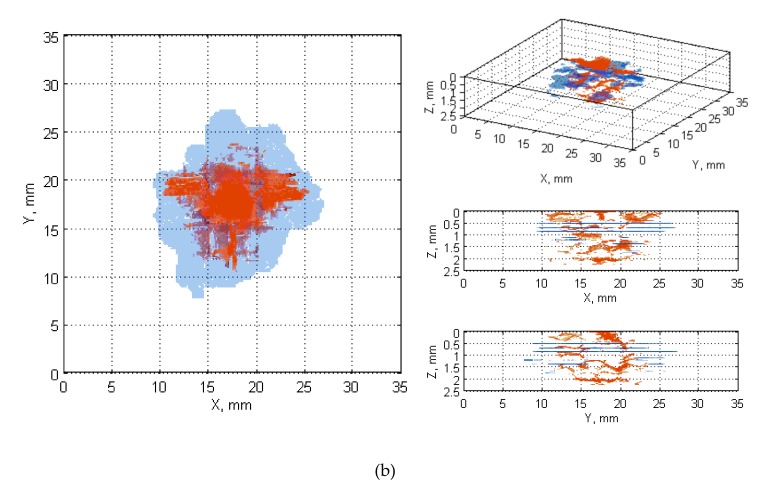
Fused damage detected in the X-ray CT scans (red color) and the ultrasonic (blue color) B-Scan (**a**) and C-Scan (**b**) for impactor D with impact energy of 12.5 J.

**Figure A10:**
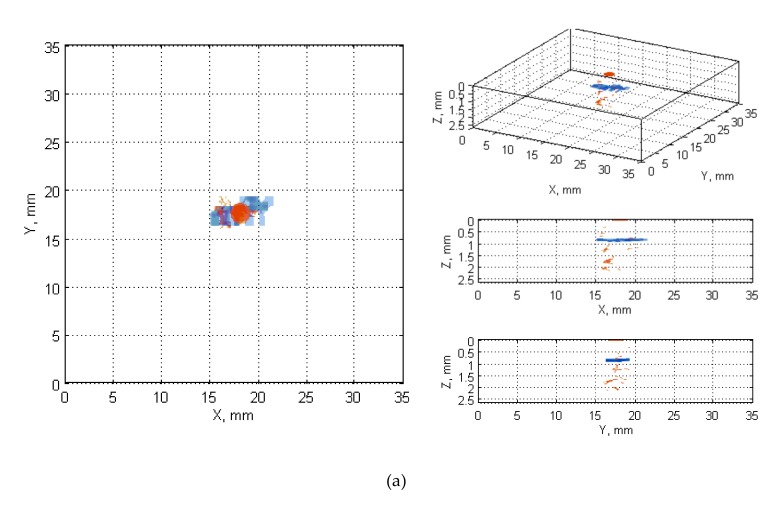

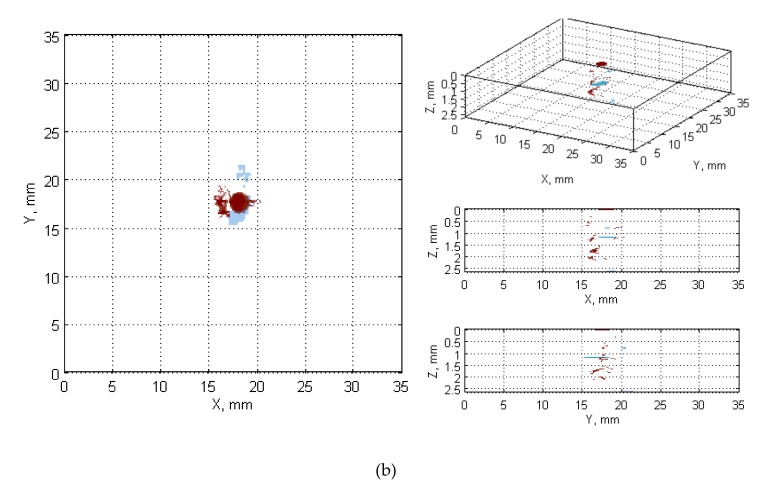
Fused damage detected in the X-ray CT scans (red color) and the ultrasonic (blue color) B-Scan (**a**) and C-Scan (**b**) for impactor E with impact energy of 5 J.

**Figure A11:**
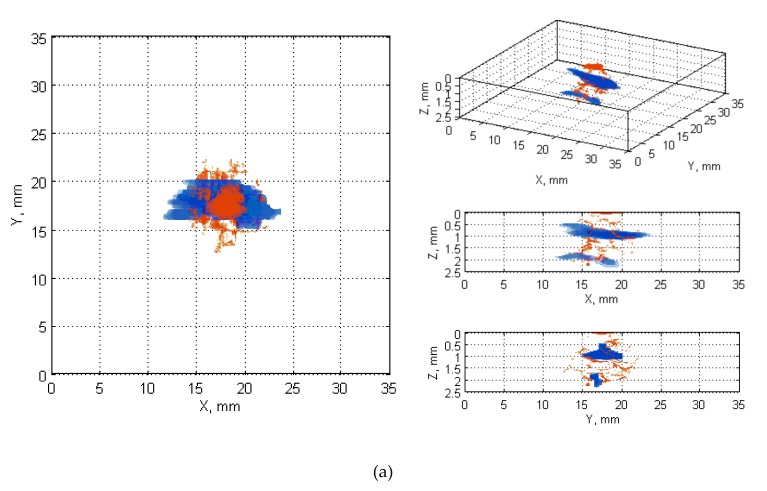

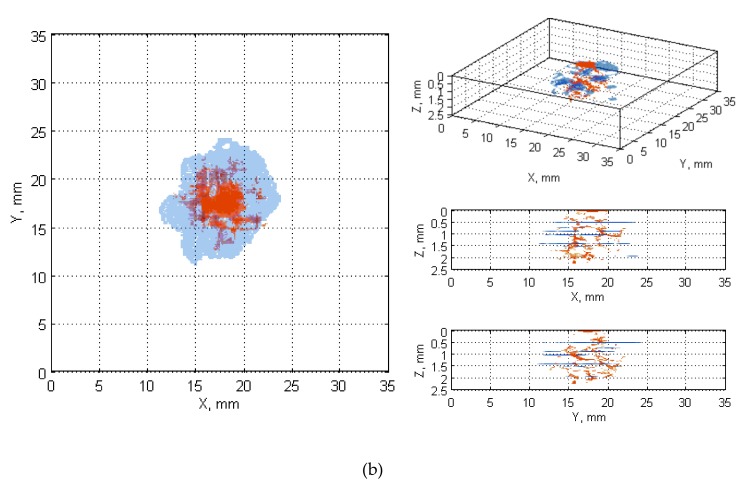
Fused damage detected in the X-ray CT scans (red color) and the ultrasonic (blue color) B-Scan (**a**) and C-Scan (**b**) for impactor E with impact energy of 7.5 J.

**Figure A12:**
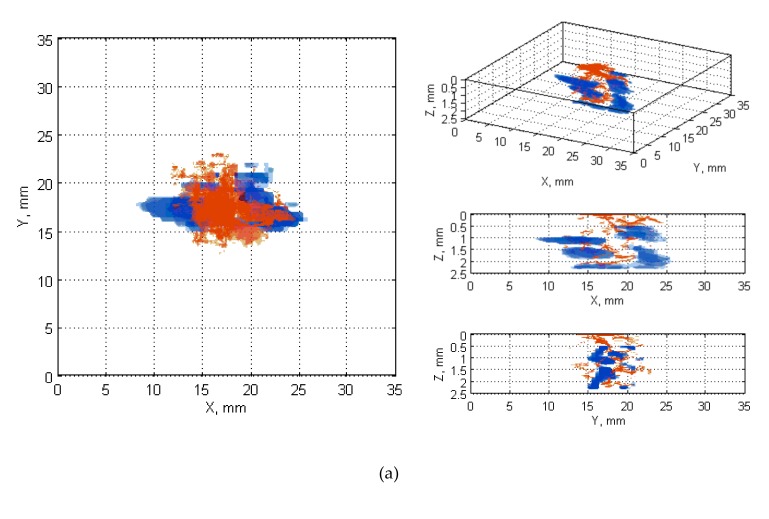

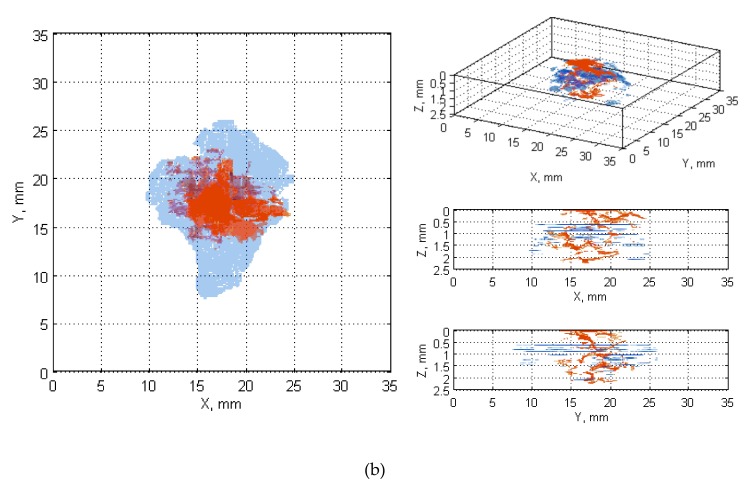
Fused damage detected in the X-ray CT scans (red color) and the ultrasonic (blue color) B-Scan (**a**) and C-Scan (**b**) for impactor E with impact energy of 10 J.

**Figure A13:**
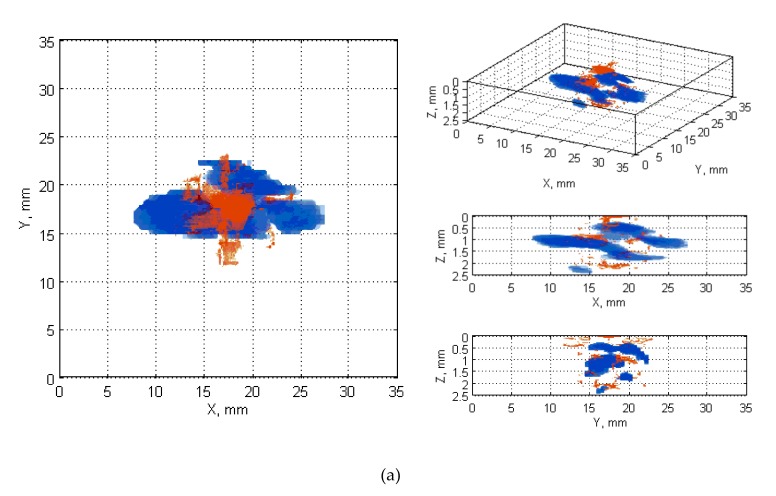

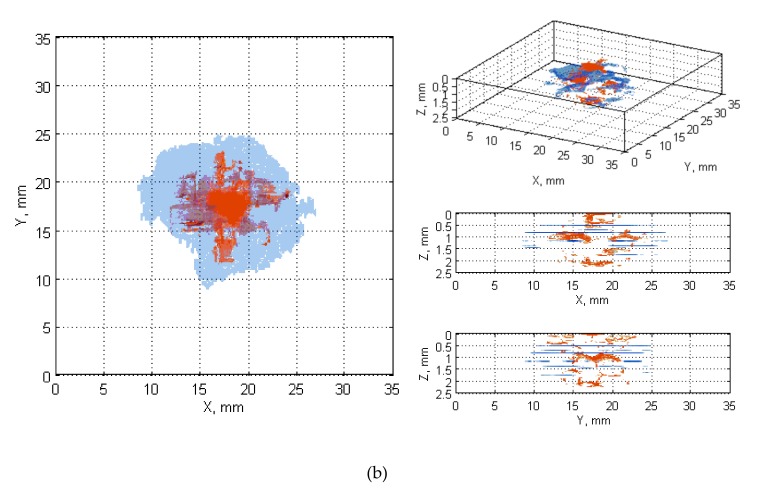
Fused damage detected in the X-ray CT scans (red color) and the ultrasonic (blue color) B-Scan (**a**) and C-Scan (**b**) for impactor E with impact energy of 12.5 J.

## Appendix A

In this appendix, the results of all processed data obtained from NDT tests for three considered impactors and corresponding impact energies in a form of fused 3D images are presented. The case E17.5 was presented in Figure 4 and Figure 5.
